# Characteristics linked to the reduction of stigma towards schizophrenia: a pre-and-post study of parents of adolescents attending an educational program

**DOI:** 10.1186/1471-2458-14-258

**Published:** 2014-03-18

**Authors:** Yiwei Ling, Mayumi Watanabe, Hatsumi Yoshii, Kouhei Akazawa

**Affiliations:** 1 Department of Medical Informatics, Niigata University Medical and Dental Hospital, Asahimachi-Dori 1-754, Niigata 951-8520, Japan; 2 Department of Health, Faculty of Health Science, Tsukuba University of Technology, Tsukuba, Japan; 3 School of Health Sciences, Faculty of Medicine, Tohoku University, Sendai, Japan

**Keywords:** Educational program, Stigma, Schizophrenia, Parents of adolescents, Multiple logistic regression

## Abstract

**Background:**

The stigma of schizophrenia constitutes a major barrier to early detection and treatment of this illness. Anti-stigma education has been welcomed to reduce stigma among the general public. This study examined the factors associated with the effectiveness of a web-based educational program designed to reduce the stigma associated with schizophrenia.

**Methods:**

Using Link’s Devaluation-Discrimination Scale to measure stigma, the effect of the program was measured by the difference in pre- and post-program tests. In the present study, we focused on program participants whose stigma towards schizophrenia had considerably improved (a reduction of three points or more between pre- and post-program tests) or considerably worsened (an increase of three points or more). The study participants were 1,058 parents of middle or high school students across Japan, including 508 whose stigma had significantly decreased after the program and 550 whose stigma had significantly increased. We used multiple logistic regression analysis to predict a considerable reduction in stigma (by three or more points) using independent variables measured before exposure to the program. In these models, we assessed the effects of demographic characteristics of the participants and four measures of knowledge and views on schizophrenia (basic knowledge, Link’s Devaluation-Discrimination Scale, ability to distinguish schizophrenia from other conditions, and social distance).

**Results:**

Participants’ employment status, occupation, basic knowledge of schizophrenia, pre-program Link’s Devaluation-Discrimination Scale score, and social distance were significant factors associated with a considerable decrease in the stigma attached to schizophrenia following the educational program. Specifically, full-time and part-time employees were more likely to experience reduced stigma than parents who were self-employed, unemployed, or had other employment status. Considerable decreases in stigma were more likely among parents working in transportation and communication or as homemakers than among other occupational groups. In addition, parents with higher pre-program levels of stigma, lower basic knowledge, or lower social distance were more likely to have reduced levels of stigma.

**Conclusions:**

Based on the regression analysis results presented here, several possible methods of reducing stigma were suggested, including increasing personal contact with people with schizophrenia and the improvement of law and insurance systems in primary and secondary industries.

## Background

The first episode or prodromal symptoms of schizophrenia typically occur in adolescence [1,2]. However, stigma towards schizophrenia is one of the most important factors related to the obstruction of its early detection and treatment [3,4]. Therefore, it is vital to decrease stigma among parents of adolescents to contribute to the removal of this barrier [5]. There have been many studies on demographic and social characteristics associated with stigma towards schizophrenia, including educational attainment, occupation, and previous contact with people with schizophrenia [4,6,7]. However, the factors that create stigma among parents of adolescents have rarely been researched. A notable exception is a recent study by Yoshii [8] that showed that the factors reducing the risk of stigma associated with schizophrenia in Japanese parents were family income, previous contact with people with schizophrenia, and participation in welfare activities for people with mental illness.

In recent decades, various educational programs have been developed aimed at reducing the stigma associated with schizophrenia. For example, a video about schizophrenia was played for 571 students from eight high schools across Canada [9]. In another example, a video-based education program was implemented among 255 students from three middle schools in Hong Kong [10]. In both cases, post-program tests found that students displayed significantly less stigma towards schizophrenia than before the program. Nonetheless, there has been no educational program specifically developed for the parents of adolescents, and hardly any studies on the factors associated with the effectiveness of educational programs. Because stigma reduction is beneficial for early detection and treatment for adolescents developing their first episode of schizophrenia, it is vital to expose parents to such programs. This study therefore evaluated the effect of a web-based educational program for parents of adolescents across Japan. Additionally, the factors associated with the effect were explored through multivariate analysis. The results should contribute to the more effective design of future educational programs.

## Methods

### Participants

The internet-based survey was conducted by a Japanese survey company. The sampling frame for this study was a total of 44,000 people listed in a database administered by this company as the parents of middle and high school students in Japan. From this group, a sub-sample of 5,000 parents was drawn using stratified random sampling (stratified by gender and place of residence). Of these parents, the 2,690 who agreed to participate in the survey answered a questionnaire about their geographical characteristics and living environment and then completed additional questionnaires about their knowledge of and views on schizophrenia, including Link’s Devaluation-Discrimination Scale for measuring stigma, both before and after watching an educational program. We refer to the tests about stigma administered before and after the program as the “pre-program test” and the “post-program test”. The interval between pre- and post-program tests was 1 week, and 2,465 parents completed the post-program test. All participants used a web-based questionnaire-answering system provided by the company conducting the survey. The present study design is a one-group pre-test and post-test design. Changes in stigma score ranging from −2 to 2 points were considered not to be significantly affected by the educational program, so the characteristics of parents with that level of change could not be used effectively to explore causal factors of educational effect. The final participants included in this study were therefore limited to the 1,058 parents whose absolute differences between pre- and post-program stigma scores were 3 points or more. Written informed consent for participation in the study was obtained from all participants. This study was approved by the Medical Ethics Committee of Niigata University.

### Demographic factors

The demographic factors and the corresponding categories used in this study are shown in Table 1. Parents’ educational attainment was split into four categories: middle/high school, vocational school, junior college, and university and graduate school [11]. Place of residence was also divided into four groups: northern region (Hokkaido/Tohoku), east-central region (Kanto/Shin-etsu/Hokuriku), west-central region (Tokai/Kinki), and western region (Chugoku/Shikoku/Kyushu/Okinawa). Employment status was divided into three categories: employed full-time, employed part-time, and other (self-employed, working in family business, or unemployed). There were seven occupational categories: production labor service, transportation and communication, sales and marketing, service industry, professional, homemaker, and other. Family income consisted of five categories: < 11,000; 11,000–32,000; 32,000–53,000; 53,000–110,000; and ≥ 110,000 US dollars [8,11].

**Table 1:** Descriptive statistics on demographic factors and stigma scales for stigma-increased and stigma-decreased groups (n = 1,058)

	**‘Stigma-increased’ group (n = 550)**		**‘Stigma-decreased’ group (n = 508)**		
	**N**	**%**	**n**	**%**	**P**
**Demographic factors**					
**Age (years) (Mean ± S.D.)**	46.4 ± 4.5		45.9 ± 4.5		0.179^a^
**Gender**					0.804^b^
Male	303	55.1	276	54.3	
Female	247	44.9	232	45.7	
**Children’s status**					0.568^b^
Junior school students	274	49.8	262	51.6	
Senior high school students	276	50.2	246	48.4	
**Education**					0.227^b^
Junior and senior high school	140	25.5	154	30.3	
Vocational school	70	12.7	69	13.6	
Junior college	80	14.5	74	14.6	
University and graduate school	260	47.3	211	41.5	
**Current residence**					0.242^b^
Hokkaido/Tohoku	59	10.7	59	11.6	
Kanto/Shin-etsu/Hokuriku	248	45.1	204	40.1	
Tokai/Kinki	180	32.7	169	33.3	
Chugoku/Shikoku/Kyushu/Okinawa	63	11.5	76	15.0	
**Married**					0.564^b^
Yes	527	95.8	483	95.1	
No	23	4.2	25	4.9	
**Family structure**					0.084^b^
Nuclear family	439	79.8	383	75.4	
Other	111	20.2	125	24.6	
**Employment status**					0.212^b^
Full-time	288	52.4	273	53.7	
Part-time	76	13.8	85	16.7	
Other	186	33.8	150	29.6	
**Occupation**					0.014*^b^
Production labor service (Mining/Construction/Manufacturing/energy industry)	137	24.9	106	20.9	
Transportation and communication	23	4.2	36	7.1	
Sales and marketing (Wholesale and retail/Finance and insurance/Real estate)	77	14.0	68	13.4	
Service industry (Catering and lodging, etc.)	58	10.6	64	12.6	
Professional (Medical and welfare/Education industry/Public officer)	93	16.9	104	20.5	
Homemaker	115	20.9	108	21.2	
Other	47	8.5	22	4.3	
**Family income per year (US dollars)**					0.733^b^
<11,000	8	1.5	9	1.8	
11,000-32,000	48	8.7	35	6.9	
32,000-53,000	98	17.8	84	16.5	
53,000-110,000	296	53.8	289	56.9	
≥110,000	100	18.2	91	17.9	
**Proximity to person with schizophrenia**					0.564^b^
Yes	21	3.8	23	4.5	
No	529	96.2	485	95.5	
**Participation in welfare activities for people with mental illness**					0.463^b^
Yes	49	8.9	52	10.2	
No	501	91.1	456	89.8	
**Factors obtained before watching the program**					
**Examination of basic knowledge (Mean ± S.D.)**	10.8 ± 1.3		10.8 ± 1.2		0.914^a^
**Pre-program Link’s Devaluation-Discrimination Scale (Mean ± S.D.)**	30.6 ± 4.2		35.8 ± 4.7		<0.001*^a^
**Examination of ability to distinguish schizophrenia from other disorders (Mean ± S.D.)**	12.0 ± 2.8		12.2 ± 2.9		0.357^a^
**Social distance scale (Mean ± S.D.)**	12.0 ± 4.5		12.5 ± 4.3		0.052^a^

^a^Mann–Whitney *U* test.

^b^Pearson’s chi-squared test.

*Two-tailed significance with P-values < 0.05.

### Measures of schizophrenia stigma and knowledge

Link’s Devaluation-Discrimination Scale modified for schizophrenia was used for measuring the stigma attached to schizophrenia among parents of middle and high school students (Additional file 1). This scale measures the extent to which a respondent believes that most people would devalue or discriminate against people with schizophrenia now or in the past [12,13]. The form of the scale attributes responses to an external population and encourages the free expression of negative attitudes. In other words, the scale is designed to reduce, to some extent, intentionally benign reactions [14]. Additionally, this scale could be completed by both normal people and those with schizophrenia [14]. The measure consists of 12 four-level Likert-type items with higher values indicating stronger stigma (1: strongly disagree, 2: tend to disagree, 3: tend to agree, 4: strongly agree). As previously mentioned, this scale was administered twice, once before and once after participants watched an educational program. Additionally, the difference between the pre- and post-program scores on Link’s Devaluation-Discrimination Scale was calculated to assess the change of stigma score.

The level of basic knowledge about schizophrenia and the ability to identify and distinguish it from other conditions were measured by two questionnaires consisting of 14 and 19 “True or False questions.” The latter scale was based on the *Diagnostic and Statistical Manual of Mental Disorders, Fourth Edition, Text Revision* criteria for schizophrenia and the PRIME Screen [9,15,16]. Social distance was evaluated by the Social Distance Scale–Japanese version, which consists of eight four-level Likert-type questions [8,11,16,17].

### Web-based educational program

An internet-based multimedia slideshow program was developed by Yoshii *et al*. and shown to parents [8,11]. The program aimed to provide basic knowledge about schizophrenia to reduce stigma. The program covered characteristics, causes, symptoms, and classification of schizophrenia as well as the course of the disease and its characteristic clinical features, treatment and prognosis, methods of preventing progression and exacerbation, signs of progression, and consultation facilities. Participants could complete this program within 13 minutes by viewing 12 slides on the same website that delivered the survey questionnaires.

### Statistical analysis

All statistical analyses were performed using IBM SPSS Statistics for Windows version 20.0 (Armonk, NY: IBM Corp.) Means (± standard deviations) were used to characterize the distributions of continuous variables. The Shapiro-Wilk test was used to assess the normality of the distribution. The Mann–Whitney *U* test was used to compare the medians in the distributions of continuous variables between groups in which stigma towards schizophrenia increased and the group where it decreased. Pearson’s Chi-squared test was conducted to test the equality of proportions among the groups. The Wilcoxon signed-rank test was used to compare the distribution of the scores between pre- and post-program tests for each item of Link’s Devaluation-Discrimination Scale. Multiple logistic regression analysis with forward stepwise model selection was used to explore the factors significantly associated with considerable changes in the stigma scale. Candidate independent variables and significant influencing factors are shown in Table 1. The dependent variable in the multiple logistic regression models concerned two events: a considerable stigma increase and a considerable stigma decrease. The stigma changes were calculated by subtracting the post-program stigma score from the pre-program stigma score for each individual. As is described above, we chose changes with an absolute value of three points or more to represent considerable changes. The logistic regression models predicted the odds of a being in the “stigma-decreased” group (changes equal to or greater than 3, coded as 1), compared with the odds being in the “stigma-increased” group (coded as 0). Nominal or ordinal variables with more than two categories were transformed to 0–1 design variables. The reference categories of these variables are described in the results for the independent variables. For the multiple logistic regression analysis, the null hypothesis is that none of the independent variables affects the probability of observing the value of 0 or 1 on the dependent variable. All statistical tests were two-tailed, and statistical significance was defined as P < 0.05.

## Results

### Participant characteristics

Table 1 shows the descriptive statistics of the participants disaggregated by “stigma-increased” or “stigma-decreased” group. The mean ages of the parents in the stigma-increased group and the stigma-decreased group (± standard deviation) were 46.4 ± 4.5 and 45.9 ± 4.5 years, respectively. The stigma-increased group was 44.9% female, and the stigma-decreased group was 45.7% female. The percentages who came from nuclear families were 79.8 and 75.4%, and the percentages who were part-time workers were 13.8 and 16.7% in the stigma-increased and stigma-decreased groups, respectively. The percentages working in the production labor service sector were 24.9 and 20.9%, the percentages working in transportation and communication were 4.2 and 7.1%, and the percentages working as professionals were 16.9 and 20.5% for those in the stigma-increased and stigma-decreased groups, respectively. The means ± standard deviation of parents’ scores on the social distance scale were 12.0 ± 4.5 and 12.5 ± 4.3 for the stigma-increased group and the stigma-decreased group, respectively.

### Distribution of pre- and post-program link’s “stigma scales”

Average scores on Link’s Devaluation-Discrimination Scale for each participant were calculated by taking the mean of scores on the 12 scale items. Based on this average stigma score, participants were divided among four stigma level categories. To describe an individual participant’s stigma level, participants were labeled as having a “minimal” (overall stigma score < 2), “low” (≥ 2 but < 2.5), “moderate” (≥ 2.5 but < 3), or “high” (≥ 3) stigma level [13,18]. Those with a minimal or low level of stigma in the pre-program test showed a tendency towards increasing stigma levels in the post-program test. Among parents with moderate and high stigma levels in the pre-program test, 24.7 and 60.0%, respectively, showed reduced levels of stigma in the post-program test. There were 321 parents who exhibited higher levels of stigma towards schizophrenia after watching the program and 331 parents who showed decreased levels. The paired distributions of pre- and post-program tests were not significantly different (Wilcoxon signed-rank test; P = 0.747) (Table 2).

**Table 2:** Change of the mean score on Link’s Devaluation-Discrimination Scale from pre-test to post-test

**Mean scores of pre-link’s devaluation-discrimination scale**	**Mean scores of post-ink’s devaluation-discrimination scale**				**Total**
	**Minimal (<2)**	**Low (2–2.5)**	**Moderate (2.5-3)**	**High (≥3)**	
**Minimal (<2)**	2 (10.0)*	10 (50.0)	7 (35.0)	1 (5.0)	20 (100.0)
**Low (2–2.5)**	15 (6.4)	41 (17.4)	168 (71.5)	11 (4.7)	235 (100.0)
**Moderate (2.5-3)**	2 (0.4)	121 (24.3)	241 (48.4)	134 (26.9)	498 (100.0)
**High (≥3)**	0 (0.0)	18 (5.9)	165 (54.1)	122 (40.0)	305 (100.0)
**Total**	19 (1.8)	190 (18.0)	581 (54.9)	268 (25.3)	1058 (100.0)

Two-tailed P-value of 0.747 for Wilcoxon signed rank-test for four-level mean scores of pre- and post-test stigma.

*The number of participants is presented in each cell, with the percent of the subtotal of each row in parentheses.

### Characteristics associated with changes of total stigma scores

To assess overall stigma changes, the means ± standard deviation of Link’s Devaluation-Discrimination Scale in pre- and post-programs for the whole sample (2,465 parents) were 32.8 ± 4.4 and 32.8 ± 4.4, respectively. In terms of the difference between pre- and post-program scores on this scale, the mean ± standard deviation was −0.06 ± 3.71. The Wilcoxon signed-rank test was used to compare the paired distributions of pre- and post-program scores on Link’s Devaluation-Discrimination Scale. The P-value of the test was 0.176, suggesting that the distributions of pre- and post-program stigma were not significantly different. What is more, of the 2,465 parents who completed both pre- and post-program tests, 994 (40.3%) had a decreased stigma score.

The results of univariate analyses for clarifying the characteristics of stigma-increased and stigma-decreased groups are also shown in Table 1. Parental occupation and pre-program score on the Devaluation-Discrimination Scale were extracted as significant factors associated with changes to the “stigma scale.” Those working in transportation and communication or the service industry, as well as professionals and homemakers, had relatively higher proportions in the stigma-decreased group. The mean total stigma score for the pre-program test in the stigma-decreased group (35.8 ± 4.7) was significantly higher than the mean score for the stigma-increased group (30.6 ± 4.2) (Mann–Whitney *U* test; P < 0.001).

Multiple logistic regression analysis was used to explore the characteristics associated with changes in total stigma scores following the educational program (Table 3). First, we estimated a logistic regression model including only demographic characteristics as independent variables (Table 3–1). Employment status and occupation were selected as significant factors associated with a considerable decrease in stigma levels. The odds ratios of full-time and part-time employment status were estimated at 1.669 and 1.897, respectively, indicating that parents in these categories were more likely to have a reduced total stigma score than those in the “other” category. Working in transportation and communication or being a homemaker also made a reduction of the total stigma score more likely. This model correctly predicted the outcome for 72.0% of the parents in the stigma-increased group and for 38.0% of parents in the stigma-decreased groups. Overall, the outcome category was correctly predicted for 55.7% of the 1,058 parents in the model (data were obtained from Classification Table output in SPSS and are not shown in the tables).

**Table 3:** Multiple logistic regression models predicting difference between pre-test and post-test scores on Link’s Devaluation-Discrimination Scale

3-1 Results of multiple logistic regression analysis using only demographic variables to predict considerably decreased stigma*						
**Variables**	**Coefficients**	**Std. error**	**P**	**Odds ratio**	**95% CI**	
					**Lower**	**Upper**
**Employment status**						
Full-time	0.512	0.224	0.022	1.669	1.077	2.587
Part-time	0.640	0.255	0.012	1.897	1.151	3.125
Other**			0.032			
**Occupation**						
Production labor service**			0.004			
Transportation and communication	0.716	0.298	0.016	2.045	1.141	3.666
Sales and marketing	0.189	0.215	0.380	1.208	0.792	1.840
Service industry	0.442	0.237	0.062	1.557	0.978	2.478
Professional	0.366	0.195	0.061	1.442	0.984	2.115
Homemaker	0.689	0.280	0.014	1.992	1.151	3.448
Others	−0.463	0.295	0.117	0.629	0.353	1.123
**Constant**	−0.752	0.246	0.002	0.472		
3-2 Results of multiple logistic regression analysis using the significant demographic variables and four measures at pre-evaluation to predict considerably decreased stigma*						
**Variables**	**Coefficients**	**Std. error**	**P**	**Odds ratio**	**95% CI**	
					**Lower**	**Upper**
**Employment status**						
Full-time	0.315	0.170	0.064	1.370	0.982	1.912
Part-time	0.564	0.231	0.015	1.758	1.117	2.767
Other**			0.037			
**Knowledge score**	−0.150	0.061	0.015	0.861	0.763	0.971
**Pre-program Link’s Devaluation-Discrimination Scale**	0.359	0.024	<0.001	1.432	1.366	1.502
**Social distance**	−0.162	0.021	<0.001	0.850	0.815	0.887
**Constant**	−8.576	0.890	<0.001	0.000		

*Dependent variable: 1 = stigma-decreased, 0 = stigma-increased. The two groups were defined by subtracting the total post-test score on Link’s Devaluation-Discrimination Scale from the total pre-test score on Link’s Devaluation- Discrimination Scale. The significant variables were selected using forward stepwise regression. Pre-evaluation measures include basic knowledge, pre-program Link’s Devaluation-Discrimination Scale score, ability to identify and distinguish schizophrenia from other conditions, and social distance.

**Reference category.

We then estimated a logistic regression model predicting a considerable reduction in stigma score including demographic factors and the four measures investigated in the pre-program test as candidate independent variables (basic knowledge, pre-program Link’s Devaluation-Discrimination Scale score, ability to identify and distinguish schizophrenia from other conditions, and social distance) (Table 3–2). These four measures showed the degree of recognition, knowledge, and stigma towards schizophrenia before watching the educational program. They were included in the model to explore the characteristics associated with stigma by investigating the link between stigma changes and the original degree of recognition, knowledge, and stigma towards schizophrenia, in addition to demographic factors. Employment status and scores on basic knowledge, pre-program score on Link’s Devaluation-Discrimination Scale, and social distance were selected as significant factors associated with a considerable decrease in total stigma score. Working part-time was significantly associated with a reduction in the total stigma score, with an estimated odds ratio of 1.758. The odds ratios for basic knowledge and social distance were estimated at 0.861 and 0.850. This means that a one-point increase in either of these variables was associated with a decrease of approximately 14–15% in the odds of having a decreased stigma score. In contrast, an increase in the pre-program Link’s Devaluation-Discrimination Scale score resulted in increased odds of a reduction of the total stigma score. The correct outcome category was predicted for 79.5% of parents in the stigma-increased group and 73.4% of parents in the stigma-decreased group. The outcome was correctly predicted for 76.6% of parents, overall (data not shown).

## Discussion

In this study, demographic and social factors were used to characterize the changes in levels of stigma towards schizophrenia, which were measured by Link’s Devaluation-Discrimination Scale before and after watching an internet-based educational program. Among parents of Japanese middle and high school students, mean stigma scores before and after watching the educational program on schizophrenia were identical (32.8 ± 4.4), and our analysis comparing the distributions indicated that the pre- and post- program distributions were not significantly different (Wilcoxon signed-rank test P-value = 0.176). The scores of pre- and post-program stigma found in the present study were similar to the stigma score reported by Berge *et al*. (32.86 ± 6.22) [19], suggesting that the result of this study measured by Link’s Devaluation-Discrimination Scale is a typical result with little fluctuation among the general population.

The design of the present study, in terms of both the delineation of the working sample and the choice of the dependent variable, facilitated the detection of new findings. Besides the parents with considerably-changed stigma scores, there were also some whose stigma scores changed slightly. These slight changes could be caused by either the educational program or random error. Consequently, if all these heterogeneous participants were studied simultaneously, it would be difficult to test the true educational effect of the program [20]. We therefore studied only those parents who showed considerably increased or decreased levels of stigma towards schizophrenia. In addition, this study is different from common multivariate analytical studies that have used stigma level as the dependent variable. Instead, we have explored the risk factors associated with the effectiveness of stigma-change through education. As a result, several little-known factors associated with stigma scores emerged, including employment status. In addition, we were able to highlight other important factors that are easily overlooked, such as differences associated with work status, occupation, and levels of basic knowledge.

In the logistic regression analysis that included only demographic and social factors as independent variables, employment status and occupation were selected as significant factors associated with considerable changes in levels of stigma (comparing the odds of considerable decreases to the odds of considerable increases). To the best of our knowledge, this is the first time that the effect of employment status has been shown on stigma towards schizophrenia. The employment status described as “other” includes those who are self-employed, working in family-owned businesses, and unemployed. The overwhelming majority of this group was unemployed (66.4%, data not shown). Compared with those with “other” employment status, levels of stigma were significantly more likely to decrease among both full- and part-time employees. The degrees of freedom of social contact are relatively high among full- and part-time employees compared with the “other” group. In particular, unemployed and self-employed people have fewer opportunities to be in contact with different kinds of people and ideas. In contrast, full- and part-time employees, such as doctors, salesmen, lawyers, and civil servants, come into contact with a wide range of people in their professional lives. This result for employment status and occupation, then, could be seen as meaning that people with wider social and interpersonal relationships were more likely to be affected by the program in terms of reducing levels of stigma towards schizophrenia. A previous study on social contact and stigma indicated that people may become broader-minded and more accepting of difference through intergroup contact, with differences going beyond age and affinity orientation. It was also reported that requiring greater interpersonal disclosure may help to reduce stigma [21].

The result on occupation type could be interpreted as a “stigma reduction” effect and a “degree of reduction difficulty” in different sectors. According to statistics of the Japanese Ministry of Health, Labour and Welfare [22], the employment rate of mentally-disabled people is significantly lower than that of physically-disabled people (0.7 vs. 19.3%). Therefore, opportunities for contact with mentally-disabled people in the workplace are likely to be very limited. In addition, 70.7% of employed mentally-disabled people engage in the tertiary industry while only 29.3% engage in other industries. This indicates that those engaged in the primary and secondary industries would have hardly any contact with mentally-disabled people. Thirty-five percent of the tertiary industry was the medical sector, and previous research has reported low stigma among doctors and nurses [23,24]. Furthermore, workplaces in the tertiary industry are often offices, while other industries sometimes provide dangerous working conditions, such as a pelagic (tuna) fishing ship, a mine, or a welding factory, all of which often involve staff security issues. As reported in a study on the relationship between stigma and barriers to employment, people in industries using heavy machinery and dangerous equipment have higher levels of stigma against mental illness because of worries about the dangers to and of employees with mental illness [25]. Therefore, the program about schizophrenia may have given the message to people engaged in these industries, with very little knowledge of or contact with mentally-disordered people, that they would have higher security needs around co-workers with mental illness. There is a possibility that their surprise and hesitation might subconsciously cause increases in stigma levels. It has been previously reported that gender differences were found in public attitudes towards mental disorder and that females showed more emotional concern about mental health problems than did males [26,27]. This might indicate that gender difference was one of the reasons why homemakers in this study, who are mostly female (96.9% homemakers were female, data not shown), showed lower levels of stigma towards schizophrenia.

Apart from demographic and social characteristics, other factors surveyed before watching the program were also used as independent variables in the logistic regression analysis. These factors included the pre-program scores on Link’s Devaluation-Discrimination Scale, basic knowledge about schizophrenia, social distance, and the ability to distinguish schizophrenia from other disorders. These measurements of schizophrenia-associated stigma obtained before the educational program were incorporated in a second model to predict the stigma changes caused by the program. Knowledge, pre-program score on Link’s Devaluation-Discrimination Scale, and social distance were included in the final logistical regression model as potentially-significant independent variables. This study demonstrated that the pre-program level of stigma was a significant explanatory variable. According to a previous study on anti-stigma education, lower levels of understanding and a more negative attitude towards mental illness allowed more potential for improvement following the program [28]. It can therefore be inferred that parents with higher stigma levels before the program are more likely to view schizophrenia more positively after watching the program. This result is shown by the logistic regression presented in Table 3–2, and also by the distribution comparison of pre- and post-program Link’s Devaluation-Discrimination Scale scores shown in Table 2.

In this study, some parents did not change their attitudes towards schizophrenia. There are two possible causes of stigma not changing. One is the increase of stigma synchronized with the improvement of knowledge about schizophrenia. In this study, increases in levels of stigma tended to occur among parents who had more knowledge before watching the program. This is consistent with some previous studies that have indicated that stigma towards mental illness increases with increased knowledge [29-32]. For example, research in 2013 by Loch *et al.* reported that the more information people were given about schizophrenia, the more negatively they viewed the illness [29]. The reason might be that many laypeople do not have the opportunity to connect with the real condition of schizophrenia. Therefore, these “knowledgeable” people actually know little about the daily life of people with schizophrenia after only reading written descriptions, and they consequently retain their original attitudes towards schizophrenia. The second possible reason is that the content of the educational program might make the audience hesitate, become discouraged, or feel hopeless because of the complexity and incomprehensibility of schizophrenia. This “side effect” of schizophrenia literacy has been confirmed by several recent studies [31-33], and might explain why levels of stigma towards schizophrenia sometimes remain unchanged or worsen following an educational program [34,35]. Specially, as parents of adolescents confront this complicated illness, associating the symptoms with negative aspects of people with schizophrenia, their fear of the illness and worries about their children may be stimulated.

The above perspectives confirm that simple knowledge-imparting programs are not always effective in decreasing stigma. Similar results were also found in Hong Kong and the United States [10,36]. Alternatively, several studies have demonstrated that interpersonal contact with stigmatized people is a recognized effective strategy for reducing public stigma, indicating that the combination of knowledge and contact should be an effective method to intensify the educational effect of programs seeking exclusively to impart knowledge [35,37].

There are several possible methods of reducing levels of stigma. In line with the above discussion about the association between employment status and stigma changes and the importance of supplementing knowledge communication with contact for parents of middle and high school students, two types of anti-stigma interventions could be conducted in the future. One involves increasing contact in daily life with people with schizophrenia. Most laypeople lack opportunities to absorb knowledge and make personal contact with people suffering from schizophrenia, especially if their contact opportunities are limited by their employment status. Suitable ways of encouraging this kind of contact might be lectures by psychiatrists and people with or recovered from schizophrenia in community centers or parents’ meetings at school. The second type of anti-stigma intervention involves incorporating contact with people with schizophrenia within the educational program itself. It is possible to include interview videos of people who suffered from schizophrenia as adolescents and their parents, who could talk about the experiences of their actual daily lives and effective treatment methods. These positive examples could make parents hopeful regarding the treatment of schizophrenia and aware of the importance of early detection and treatment. Furthermore, improving the law and insurance systems in primary and secondary industries could make people more willing and relaxed regarding the possibility of working with people with schizophrenia, thus reducing the stigma among this population [38-40].

This study also has several limitations, and some of the conclusions drawn here will lead to further debate. First, because the invitation to take part in the survey and the viewing of the educational program were conducted over the internet, parents who frequently used the internet were more likely to be included in the sample. Therefore, the sample in this study may not be representative of the wider population because of the nature of internet-based surveys, which makes the occurrence of non-response inevitable. Of the 5,000 candidates for participation in this study, 2,310 parents did not respond. For these parents, we were unable to obtain agreement to participate or any data regarding their characteristics, and it is possible there are some differences between respondents and non-respondents in terms of characteristics and attitudes. Second, regional cultural and psychological factors were not considered as explanatory variables of the effects. These would be a valuable consideration future studies. Third, the result of this study, with almost equal numbers of participants in the stigma-increased (n = 550) and stigma-decreased (n = 508) groups, underlines the fact that the theory of how educational efforts can affect stigma changes is in need of further development. However, the primary objective of this study was to explore the factors related to the characterization of considerably decreasing or increasing stigma using appropriate statistical analyses, and the results suggest that our educational program resulted in considerable decreases in stigma for participants with specific characteristics. This finding has encouraged us to conduct further studies. Concretely, we need to evaluate the reproducibility of the findings on the stigma-decreasing effect of our educational program for the Japanese parents of adolescents with certain characteristics. Additionally, it is necessary for us to investigate the cause of increasing stigma for the other parents in detail and to consider whether there are alternative strategies to prevent the considerable increases in stigma observed among some parents in this study. In particular, it is essential that future research give more deliberate consideration to strategies for anti-stigma education for parents with a variety of characteristics.

## Conclusions

The results of this study suggest that participants’ employment status, occupation, basic knowledge of schizophrenia, initial Link’s Devaluation-Discrimination Scale score, and social distance were significant factors associated with the likelihood of a considerable decrease in stigma towards schizophrenia as a result of the educational program. The results of the analysis presented in this article have several implications in terms of promising methods for future anti-stigma education efforts. For example, contact with people with schizophrenia in daily life would likely be effective for parents whose interpersonal contacts are limited, video contact with adolescents with schizophrenia and their parents might encourage hopefulness and awareness regarding schizophrenia among parents, and the improvement of the law and insurance systems would intensify the sense of security of parents working in primary and secondary industries, consequently decreasing the fear and stigma associated with schizophrenia.

## Competing interests

The authors declare that they have no competing interests.

## Authors’ contributions

YL was the principal investigator and was responsible for the study concept and design, was involved in the data management, carried out the statistical analyses, and drafted the manuscript. MW was involved in drafting the manuscript. HY conducted the data collection and was involved in the data management. KA supervised the first author and was involved in interpreting the data and drafting the manuscript. All authors revised the text critically for important intellectual content and read and approved the final manuscript.

## Pre-publication history

The pre-publication history for this paper can be accessed here:

http://www.biomedcentral.com/1471-2458/14/258/prepub

## Supplementary Material

Additional file 1 The Link Devaluation–Discrimination Scale (modified for schizophrenia) (4: strongly agree, 3: tend to agree, 2: tend to disagree, and 1: strongly disagree). Click here for file
