# Supplementary material for: Characteristics linked to the reduction of stigma towards schizophrenia: a pre-and-post study of parents of adolescents attending an educational program
Source: BMC Public Health. 2014 Mar 18;14:258. doi: 10.1186/1471-2458-14-258 (PMC4000132; doi:10.1186/1471-2458-14-258)
Supplement: Additional file 1 — The Link Devaluation–Discrimination Scale (modified for schizophrenia) (4: strongly agree, 3: tend to agree, 2: tend to disagree, and 1: strongly disagree). [file 1471-2458-14-258-S1.docx]

The Link Devaluation–Discrimination Scale (modified for schizophrenia) (4: strongly agree, 3: tend to agree, 2: tend to disagree, and 1: strongly disagree)

| Q1 | Most people would accept a person with schizophrenia as a close friend. |
| --- | --- |
| Q2 | Most people believe that a person with schizophrenia is as intelligent as the average person. |
| Q3 | Most people believe that a person with schizophrenia is just as trustworthy as the average citizen. |
| Q4 | Most people would accept a person who had fully recovered from schizophrenia as a teacher of young children in a public school. |
| Q5 | Most people feel that suffering from schizophrenia is a sign of personal failure. |
| Q6 | Most people would not hire a person with schizophrenia to take care of their children, even if he or she had been well for some time. |
| Q7 | Most people think less of a person who has had schizophrenia. |
| Q8 | Most employers will hire a person with schizophrenia if he or she is qualified for the job. |
| Q9 | Most employers will pass over the application of a person with schizophrenia in favor of another applicant. |
| Q10 | Most people in my community would treat a person with schizophrenia just as they would treat anyone. |
| Q11 | Most young women would be reluctant to date a man who has schizophrenia. |
| Q12 | Once they know a person had schizophrenia, most people will take his or her opinions less seriously. |

Q1, 2, 3, 4, 8, and 10 were reversed items and were reverse-scored.
